# The Hebrew version of the adolescent and adult time inventory–time attitudes scales (AATI–TA): a validation study

**DOI:** 10.1038/s41598-023-39431-9

**Published:** 2023-07-28

**Authors:** Eliane Sommerfeld, Zena R. Mello, Frank C. Worrell

**Affiliations:** 1 Department of Psychology, Ariel University, Ariel, Israel; 2 San Francisco State University, San Francisco, USA; 3 University of California, Berkeley, USA

**Keywords:** Psychology, Human behaviour

## Abstract

In the present study, we examined the construct validity of scores on the Hebrew version of the Adolescent and Adult Time Inventory–Time Attitudes Scale (AATI–TA). The AATI–TA was translated into Hebrew by bilingual speakers, using the back-translation method. Participants included 452 young adults (*M*_*age*_ = 22.47, *SD*_*age*_ = 1.98, 51.5% female). Several measures of psychological well-being were used to establish convergent validity, including scales assessing self-esteem, life satisfaction, and optimism, and depressive, anxiety, and somatic symptoms. Internal consistency estimates for AATI–TA scores were high and confirmatory factor analyses supported the six-factor structure. Correlational analyses indicated the expected relationships between time attitudes and measures of psychological well-being, providing convergent validity support for the AATI–TA scores. The findings of this study support the use of the Hebrew version of the AATI–TA in further studies about time attitudes.

## Introduction

Time perspective has been implicated in psychological functioning for several decades. Lewin^1^ argued that time perspective represented the “totality of the individual’s views of his psychological future and psychological past existing at a given time” (p. 75) with broad implications for psychological functioning, and Zimbardo and Boyd^2^ contended that time perspective consists of emotional, cognitive, and social components. Despite the broad conceptualization of time perspective^1,2^, much of the research on time constructs has focused on beliefs or cognitions about the future (e.g., hope, optimism) and their role in adaptive functioning^3,4^. Several years ago, Mello and Worrell^5^ introduced a multidimensional model of time perspective assessing several time perspective constructs, including time meaning (how individuals define the time periods), time frequency (how often people think about the time periods), time orientation (which time periods are perceived as the most important by individuals), time relation (how people see the time periods as related), and time attitudes (the ways individuals feel about the three time periods).

Attitudes towards time represent the emotional aspect of time perspective, namely positive and negative feelings about the past, present, and future. The Adult and Adolescent Time Inventory–Time Attitudes Scale (AATI–TA)^6^ is a self-report instrument developed to assess time attitudes. It consists of six subscales—Past Positive, Past Negative, Present Positive, Present Negative, Future Positive, and Future Negative—and AATI–TA items include both general references to the three time periods as well as specific references to the past (e.g., childhood), the present (e.g., my life right now), and the future (e.g., thinking ahead). The 6-factor structure has consistently yielded the best fit and AATI–TA scores have been found to be associated with a range of important constructs^7^. It has been translated into several languages and AATI–TA scores have been found to predict outcomes in several domains, including education^8^ and health-related behaviors^9^ in several different countries. The AATI–TA is the most frequently used subscale in this model^5^.

However, the AATI–TA has not yet been translated into Hebrew. The aim of the present study was to create a Hebrew version of the AATI–TA, to examine the internal consistency and structural validity of these scores, and to determine how they are related to psychological well-being in this national context. The Hebrew version of AATI–TA will make it possible to investigate attitudes towards time in the Israeli population, while providing important insights into the cultural, social and psychological aspects of Israeli society while making cross-cultural comparisons.

Attitudes toward time have been associated with a variety of psychological constructs related to well-being in adolescents^7,8,10^, and adults^11,12^. For example, Worrell and Andretta^7^ reported that positive attitudes toward their past, present, and future are positively and meaningfully related to hope (− 0.21 ≤ *r* ≤ 0.47) and optimism (− 0.47 ≤ *r* ≤ 0.44), whereas negative attitudes have negative relationships with these constructs. Moving beyond bivariate, variable-centered analyses to person-centered analyses, Worrell and Andretta^7^ also noted that time attitude profiles made up of the six time attitudes and obtained from cluster analyses and latent profile analyses have stronger associations with outcomes than the individual time attitude scores. Similarly, Cole et al.^11^ have found that adults with negative time attitude profiles had meaningfully higher depressive and anxiety symptoms than those with positive profiles. Time attitude scores and profiles have also been found to be associated with self-efficacy, perspective-taking, and life satisfaction in Germany^13^, and self-esteem and perceived stress in the United States^8^.

The AATI–TA has six subscales assessing positive and negative attitudes towards the past, present, and future. The AATI–TA has already been translated into several languages other than English (e.g., Albanian, Amharic, German, Italian, Japanese, Korean, Polish, Slovene, and Spanish), and based on a recent meta-analysis, scores on the six factors have been found to be psychometrically robust in a large number of studies^14^. The aim of the present study was to examine the internal consistency, structural validity, and convergent validity of the Hebrew translation of the AATI–TA in a large sample of adults. To examine convergent validity, three indicators of mental health, which are used in many studies as indicators of psychological distress in the general population, were chosen as criteria^12,14,15^: depression, anxiety, and somatization. In addition, three indices of psychological well-being were selected: self-esteem, optimism, and life satisfaction. Our hypotheses were that (a) scores on the six factors would be internally consistent, (b) the six-factor structure would be supported, and (c) AATI–TA scores would be meaningfully associated with depressive, anxiety and somatic symptoms, as well as with self-esteem, life satisfaction, and optimism. Specifically, we expected that positive time attitudes would be negatively correlated with depression, anxiety and somatization and positively correlated with self-esteem, life satisfaction, and optimism, and that negative time attitudes would show the inverse pattern.

## Methods

### Participants

The sample consisted of 452 young adults (51.5% female) from all regions of Israel, who responded to the invitation to participate, through a professional survey company, The Midgam Project Web Panel (www.midgampanel.com). The appeal was made to young people in emerging adulthood, who were 18–25 years old (*M* = 22.47, *SD* = 1.98). More than half the participants (56.4%) reported being single, and the others were married (42.9%). Two participants reported being separated or divorced. The majority (72.8%) reported having no children, and the remaining had one child (15%), two children (9.1%), or three children or more (2.8%). Most of them had post-secondary education or a bachelor's degree (63.1%), whereas others had only a high school education or below (36.1%). Almost half of the participants were employees (48.2%), others were students (37%), self-employed (4%), working in management positions (3.8%), unemployed (3.8%), or soldiers (3.1%).

### Measures

#### Adolescent and adult time inventory–time attitudes (Hebrew version)

The AATI–TA^6^ was designed to assess attitudes toward time. It has six subscales with five items in each scale—Past Positive, Past Negative, Present Positive, Present Negative, Future Positive, and Future Negative—resulting in a total of 30 items. Examples of items include “I have happy thoughts about my past” (Past Positive), “My past is a time in my life that I would like to forget” (Past Negative), “Overall, I feel happy about what I am doing right now” (Present Positive), “My current life worries me” (Present Negative), “I look forward to my future” (Future Positive), and “Thinking about my future makes me sad” (Future Negative). Responses to the items are on a 5-point scale (1 = *Totally Disagree*, 3 = *Neutral*, 5 = *Totally Agree*). Higher scores indicate a greater endorsement of a particular item and subscale scores consist of the averages of the five items on each subscale. Thus, scores on the positive items are inversely related to scores on the negative items.

There is strong psychometric evidence supporting the construct validity of AATI–TA scores^9–14,16,17^. Internal consistency estimates have typically been > 0.75, with some exceptions for Future Negative scores, and the six-factor structure has been supported in several languages. The translation process of the inventory used in this study included an initial translation from English to Hebrew by two bilingual individuals. The two Hebrew versions were compared, and differences were discussed and resolved. Then, the scale was back translated to English, and differences in wording were resolved by a bilingual translator.

#### Psychological distress

The PHQ consists of a series of self-report questionnaires designed to assess various mental health conditions^18^. PHQ questionnaires were used to assess depression, anxiety, and somatic symptoms.

##### Depression

Depression was assessed with the Patient Health Questionnaire (PHQ-9)^19^. The PHQ-9 is one of the modules within the PHQ that focuses on assessing levels of depressive symptoms, and it is a stand-alone 9-item screening scale. Participants are asked to report how often they were bothered by nine symptoms of depression over the last two weeks on a four-point scale (0 = *not at all* to 3 = *nearly every day*), for instance “little interest or pleasure in doing things.” The internal consistency estimate for scores in this sample was good (α = 0.87). Scores were calculated as a sum of the items’ ratings, with higher scores indicating a higher level of depression.

##### General anxiety

Anxiety was assessed by the General Anxiety Disorder-7-Item Scale (GAD-7)^20^. The GAD-7 is a screening scale that was also developed in the context of the PHQ. It is a 7-item self-report questionnaire that assesses general anxiety symptoms. Participants are asked to report how often they were bothered by seven symptoms of general anxiety over the last two weeks on a four-point scale (0 = *not at all* to 3 = *nearly every day*), for example “feeling nervous, anxious or on the edge.” In the present study, the internal consistency estimate for GAD-7 scores was good (α = 0.90). Scores were calculated as a sum of the items’ ratings, with higher scores reflecting higher levels of anxiety.

##### Somatic symptoms

The presence and severity of somatic symptoms were assessed by the Patient Health Questionnaire (PHQ-15)^21^. This is a specific module within the PHQ that comprises of 15 items, designed to assess somatic symptoms’ burden and severity. One item refers to problems related to the menstrual cycle and is intended for women only. Participants are asked to report how often they were bothered by somatic symptoms (e.g., headaches, stomachache) over the last four weeks on a three-point scale (0 = *not bothered me at all* to 2 = *bothered me very much*). The internal consistency index for scores was good (α = 0.84). As the number of items is different between men and women, a general score was calculated based on the average of the ratings in the items, with higher scores indicating more severe somatic symptoms.

#### Psychological well being

##### Self-esteem

Global self-esteem was assessed by the Rosenberg Self-Esteem Scale^22^. Participants are asked to rate 10 items on a four-point scale (0 = *strongly disagree* to 3 = *strongly agree*). An example of an item is “On the whole, I am satisfied with myself.” The internal consistency estimate for self-esteem scores was good (α = 0.88). A total score was calculated as a sum of the item ratings, with higher scores reflecting higher levels of self-esteem.

##### Satisfaction with life

Satisfaction with life was assessed by the 5-item Satisfaction with Life Scale^23^. Participants were asked to rate the items on a five-point scale (1 = *strongly disagree* to 5 = *strongly agree*). An example of an item is “If I could live my life over, I would change almost nothing.” The internal consistency estimate for scores in this sample was good (α = 0.87). Total scores were calculated as a sum of all items, with higher scores reflecting higher levels of satisfaction with life.

##### Optimism

Optimism was assessed by the Life Orientation Test—Revised^24^. This 10-item measure assesses dispositional optimism vs. pessimism. From the 10 items, three items measure optimism (e.g., “Overall, I expect more good things to happen to me than bad”), three items measure pessimism (e.g., “If something can go wrong for me, it will”), and four items serve as fillers and are not scored. Items are rated on a 5-point scale (1 = *strongly disagree* to 5 = *strongly agree*). After reversing the pessimism items, the internal consistency index of the 6 items was good (α = 0.79), so a total mean score was calculated, with higher scores indicating higher levels of optimism.

### Procedure

The study was approved by the Institutional Review Board of Ariel University and was performed in accordance with relevant guidelines and regulations. Informed consent was obtained from all individual participants included in the study. Data collection took place through Qualtrics (Qualtrics, Provo, UT), which is an online platform for survey data collection.

## Results

Table 1 contains the means and standard deviations for the major variables in the study. As can be seen, the means for the negative time attitude subscales were on average lower than for the positive subscales, which is a typical pattern with these scores^14^. Differences were statistically significant (*p* < 0.001) and yielded substantial effect sizes (*d*s > 1.5). Correlations among the time attitude subscales are presented in Table 2. Also as is typical, correlations within a time period (e.g., past positive and past negative =  − 0.74) are stronger than correlations between two time periods (e.g., past positive and present positive = 0.55, past negative and past positive = − 0.52, *z* = − 19.05, *p* < 0.001).

**Table 1:** Means and standard deviations of and intercorrelations among major constructs.

Time attitudes *M* (*SD*)	Depression	Anxiety	Somatization	Self-esteem	Life satisfaction	Optimism
*M*	14.96	11.08	1.39	21.14	17.54	3.62
*SD*	(5.17)	(4.32)	(0.33)	(5.83)	(4.22)	(0.82)
Past-positive 3.70 (0.87)	− 0.32*	− 0.28*	− 0.17*	0.35*	0.53*	0.38*
Past-negative 2.15 (0.97)	0.42*	0.40*	0.26*	− 0.46*	− 0.53*	− 0.37*
Present-positive 3.70 (0.82)	− 0.52*	− 0.44*	− 0.27*	0.55*	0.78*	0.46*
Present-negative 2.25 (0.92)	0.58*	0.51*	0.30*	− 0.58*	− 0.72*	− 0.50*
Future-positive 3.86 (0.76)	− 0.32*	− 0.26*	− 0.15	0.45*	0.50*	0.44*
Future-negative 1.79 (0.74)	0.49*	0.38*	0.28*	− 0.60*	− 0.51*	− 0.52*
Depression	–					
Anxiety	0.75*	–				
Somatization	0.57*	0.54*	–			
Self-esteem	− 0.55*	− 0.48*	− 0.34*	–		
Life satisfaction	− 0.49*	− 0.41*	− 0.28*	0.53*	–	
Optimism	− 0.45*	− 0.43*	− 0.26*	0.56*	0.48*	–

*N* = 452.

*p < 0.001.

**Table 2:** Correlation matrix for the 30-item adolescent and adult time inventory—time attitudes (Hebrew version).

	1	2	3	4	5	6
1. Past positive	1.00	**− 0.84**	**0.62**	**− 0.60**	**0.39**	**− 0.50**
2. Past negative	− 0.74	1.00	**− 0.59**	**0.70**	**− 0.38**	**0.58**
3. Present positive	0.55	− 0.52	1.00	**− 0.94**	**0.62**	− **0.65**
4. Present negative	− 0.52	0.61	− 0.85	1.00	**− 0.62**	**0.79**
5. Future positive	0.33	− 0.31	0.53	− 0.52	1.00	**− 0.78**
6. Future negative	− 0.38	0.48	− 0.54	0.64	− 0.65	1.00

*N* = 452. Correlations below the diagonal are for the observed variables. Bolded correlations above the diagonal are for latent variables and were obtained from the 6-factor model of the confirmatory factor analysis.

The pattern of correlations among the validation variables (see Table 1) was also as expected, with positive intercorrelations among (a) depression, anxiety and somatization and (b) self-esteem, life satisfaction, and optimism. As expected, the correlations between the mental distress and mental well-being variables were negative. The pattern of correlations among the validation variables is important in setting the stage for their use in validating time attitude scores, as these variables need to be functioning as expected for them to be useful as criterion variables for establishing external validity.

### Confirmatory factor analyses

As the AATI–TA is based on an established theoretical model^1^, which has been supported in several national contexts and developmental periods^8–14,16,17,25^, we used confirmatory factor analyses (CFAs) to examine model fit of the factor solution for AATI–TA Hebrew scores. CFAs are also appropriate for assessing AATI–TA scores given the high intercorrelations between subscales assessing the same time period^17^. All CFA models were analyzed with Mplus 8.1.5^26^ using the weighted least squares robust (WLMSV) estimator recommended for use with ordinal data^27^. Goodness of fit was evaluated with the comparative fit index (CFI), Tucker-Lewis index (TLI), and root mean square error of approximation (RMSEA). As with previous studies of the AATI–TA, we accepted values ≥ 0.90 for the CFI and TLI as acceptable and ≥ 0.95 as excellent^28,29^. For the RMSEA, values ≤ 0.08 were considered acceptable and values ≤ 0.05 were considered excellent^28,29^. These fit indices are more appropriate for scales that are based on item-level indicators with multiple factors^28^.

As in the original study introducing the AATI–TA, we tested the three theoretically viable CFA models: a two-factor model representing positive and negative attitudes (valence), a three-factor model representing past, present, and future attitudes (time periods), and the proposed six-factor model, which has been supported in previous studies^14^. As can be seen in Table 3, fit indices for the two-factor model were below the acceptable threshold. The three-factor model based on time periods yielded a CFI value in the excellent range, a TLI value in the acceptable range, and a RMSEA value in the poor range. In contrast, the six-factor model yielded CFI and TLI values in the excellent range and an RMSEA value in the acceptable range. We concluded that these results support the use of the 6-factor structure of the AATI–TA Hebrew scores as in previous studies^16,17^. Factor coefficients for the subscale items, which are presented in Table 4, ranged from 0.53 to 0.94, indicating strong loadings on the factors. Alpha and omega reliability estimates based on the coefficients were substantial (see Table 4). Correlations among the latent constructs were in keeping with theory and the results of past studies (see Table 2).

**Table 3:** Fit indices for the AATI–TA-Hebrew scores derived from confirmatory factor analyses (weighted least squares robust).

Model	*χ*^2^	*df*	CFI	TLI	RMSEA (90% C.I.)
1. Null	28,663.53	435			
2. 2-Factor (valence)	4972.80*	404	0.838	0.826	0.158 (0.154, 0.162)
3. 3-Factor (time periods)	1787.00*	402	0.951	0.947	0.087 (0.083, 0.091)
4. 6-Factor (theorized)	1073.33*	390	0.976	0.973	0.062 (0.058, 0.067)

*N* = 452.

*AATI–TA* adolescent time inventory–time attitudes scale, *CFI* comparative fit index, *TLI* Tucker–Lewis index, *RMSEA* root mean square error of approximation, *C.I.* confidence interval.

*p < 0.001.

**Table 4:** Standardized coefficients for six-factor Hebrew ATTI-TA items.

Factors	Standardized coefficients	Effect size (*r*^2^)	Factors	Standardized Coefficients	Effect size (*r*^2^)
Past positive (α = 0.90, ω = 0.93)			Past negative (α = 0.91, ω = 0.95)		
Items			Items		
3	0.83	0.68	6	0.82	0.67
9	0.85	0.72	12	0.88	0.77
21	0.86	0.73	15	0.91	0.82
24	0.82	0.67	18	0.84	0.70
30	0.91	0.83	27	0.90	0.81
Present positive (α = 0.92, ω = 0.95)			Present negative (α = 0.90, ω = 93)		
Items			Items		
5	0.91	0.82	2	0.88	0.77
11	0.89	0.79	8	0.83	0.68
14	0.92	0.84	20	0.88	0.77
17	0.80	0.64	23	0.86	0.73
26	0.88	0.77	29	0.77	0.59
Future positive (α = 0.87, ω = 0.90)			Future negative (α = 0.80, ω = 0.87)		
Items			Items		
1	0.60	0.36	4	0.83	0.68
7	0.87	0.75	10	0.77	0.59
13	0.94	0.88	16	0.88	0.77
19	0.81	0.65	22	0.74	0.54
28	0.80	0.64	25	0.53	0.28

Omega (ω) estimates are based on the factor coefficients.

### Convergent validity analyses

The correlations between scores on the time attitude and validation subscales are presented in Table 1. The correlations between the AATI–TA scores and the other psychological measures were in keeping with hypotheses. Specifically, positive attitudes toward the past, present, and future were significantly associated with lower levels of depression and anxiety and higher levels of self-esteem, satisfaction with life, and optimism, and positive attitudes toward the past and present were significantly associated with lower levels of somatization. Additionally, negative attitudes toward the past, present, and future were significantly associated with higher levels of depression, anxiety and somatization, and with lower levels of self-esteem, satisfaction with life, and optimism. Using Ferguson’s^30^ recommendations for effect size interpretation, 14 of the 36 correlations (39%) were moderate in size, and 34 of the 36 (94%) met the criteria for interpretation. As can be seen, somatization produced the lowest correlation indices; the strongest relationships found were between life satisfaction and positive and negative attitudes toward the present (see Table 1).

## Discussion

In the present study, we examined the psychometric properties of the Hebrew version of the AATI–TA—including internal consistency, structural validity, and convergent validity—in a sample of young adults in Israel. The results indicate that Hebrew AATI–TA scores are robust. Similar to other studies with the original scale, as well as with its translation into different languages^9,31^, the six-factor structure of the Hebrew version of the AATI–TA was confirmed, and its six scores, reflecting negative and positive attitudes toward the past, present, and future, were found to be meaningfully related to measures of mental distress and well-being. Specifically, positive attitudes toward time were associated with lower levels of depression and anxiety, and to a lesser extent lower levels of somatic symptoms. Positive time attitudes were also associated with higher levels of self-esteem, life satisfaction, and optimism. These findings are in keeping with other studies^6,10,11^ in which researchers have reported similar results.

The positive associations between depression and negative attitudes toward the past, present, and future can be understood in the context of cognitive models of depression. Specifically, in his seminal work, Beck^32^ postulated that depression is a result of a negative cognitive triad, that is, a negative view of the world, of the self, and of the future. Roepke and Seligman^33^ claimed that the crucial element of the cognitive triad is *faulty prospection*, that is, negative future thinking, as people with a negative view of themselves and the world could avoid depression if they held on to the notion that the future would be better. These ideas are in line with previous studies that have shown that depression is associated with hopelessness^34^ or with cognitive biases that increase the accessibility of negative memories and hence also make it difficult to construct positive scenarios for the future^35^. Indeed, depression is especially connected with a pessimistic attitude toward the future^36^, faulty prospection^33^, and hopelessness^34^, and it is also related to a recollection of negative memories^37^ and failing to accept the past^38,39^.

In addition, the evaluation of the present is also affected, as depression is related to a reduced ability to enjoy life's pleasures and lack of interest in activities. Indeed, a qualitative study revealed that depressed people tend to experience their present as out of their control, meaningless, and related to negative emotions^40^. Other quantitative studies^41,42^ revealed that depression and low life satisfaction were associated with a low perception of control in life and with a perception that things happen by chance, regardless of one's efforts. Depressed mood was found to be associated also with preoccupation with the negative qualities of the self, which in turn may further stimulate a depressed mood^43–45^. In addition, depressed individuals were prone to rumination about their past or present distress^46^.

In the present study, anxiety had a similar pattern of relationships with attitudes toward time as depression. Greater anticipation of negative experiences and reduced anticipation of positive experiences can manifest as anxiety about the future^47^, or as maladaptive worry^48^. However, the current results indicate that anxiety is also related to the way people evaluate their past and their present. These findings are in keeping with the findings of Cole et al.^11^, who reported associations of anxiety with past positive and present negative attitudes. Cognitive bias may also account for these associations. Anxious individuals tend to pay more attention to threatening stimuli^49^, and when they are asked to recall negative autobiographical memories, their memories are stronger and more vivid, they experience physical sensations similar to those they experienced in the original event, and they also tend to experience stronger negative emotions, compared to non-anxious people^50^.

Importantly, time attitudes are also strongly related to self-esteem, optimism, and life satisfaction. These findings are consistent with studies that have shown a connection between high self-esteem and a tendency to experience more positive emotions and less negative emotions^51^, as well as quality of life^52^. Dispositional optimism was also found to be associated with effective coping, health, and well-being^53^, showing that optimism is not only about positive expectations to the future, but a more general tendency to positive coping and experiencing of life. Given the association between optimism and time attitudes is < 0.50^7^, indicating less than 25% shared variance, it is likely that time attitudes and optimism make independent contributions to health and wellbeing, a finding that should be explored in subsequent studies. The findings in the current study point to the relationships among mental well-being, healthy dispositions, and attitudes toward time, and also indicate that the Hebrew version of the AATI–TA produced indicators that behave as expected based on what is known about the relationship among these constructs, supporting the validity of inferences from this instrument’s scores.

## Limitations and conclusion

There were several limitations in the current study that require caution in drawing conclusions. The sample was relatively large, but the sampling method does not allow us to conclude that the sample is representative of the young adult Israeli population. Moreover, adolescents and older adults were not included in the sample, Therefore, more research is needed before we can claim that the AATI–TA is useful in studying time attitudes in all Israelis. Moreover, the study is cross-sectional, so no causal inferences can be drawn from the findings. In addition, all variables were assessed through self-report measures, and so no inferences can be made from the present findings on the associations between time attitudes and depressive or anxiety clinical disorders. Finally, this is the first validation study of the Hebrew version of the AATI–TA^54^. Further studies are needed in order to replicate our findings. Even so, scores on the Hebrew version of the AATI–TA in the present study yielded very good psychometric indices and provide support for it to be used with Hebrew-speaking samples in future studies on attitudes towards time.

## Data Availability

The datasets generated and analyzed during the current study are not publicly available due to privacy restrictions, in accordance with the requirements of the IRB, but are available from the corresponding author on reasonable request.
